# Overweight and obesity by school socioeconomic composition and adolescent socioeconomic status: a school-based study

**DOI:** 10.1186/s12889-021-11752-2

**Published:** 2021-10-11

**Authors:** Maxime Luiggi, Olivier Rey, Maxime Travert, Jean Griffet

**Affiliations:** 1 Aix Marseille Univ, ADEF, Marseille, France; 2 Structure Fédérative d’Études et de Recherches en Éducation de Provence, FED 4238, Marseille, France; 3 Aix Marseille Univ, CNRS, ISM, UMR 7287, Marseille, France

**Keywords:** Weight status, Socioeconomic status, Socioeconomic composition, Physical activity, Lunch type

## Abstract

**Background:**

The main objective of this study was to investigate the interaction effect of school socioeconomic composition (SEC) and adolescent socioeconomic status (SES) in the prevalence of overweight and obesity among a representative sample of French adolescents of the third most populous *département* of France.

**Methods:**

1038 adolescents agreed to participate (response rate: 91.4%). They self-reported anthropomorphic variables, SES, school lunch and physical activity. The body mass index was divided into six categories according to the Center for Disease Control. Multivariable binary logistic regressions analysis without and with interaction term were performed on overweight or obesity. Models fit was compared using the Aikaike Information Criterion. Odds-ratios (OR) and their 95% accelerated-bootstrap confidence interval (95%BCa CI) were computed to estimate overweight or obesity risk.

**Results:**

8.9% of the adolescents were overweight. 3.4% were obese. No school-SEC effect was observed among low-SES adolescents. Medium-SES adolescents were at greater risk in low-SEC (OR = 10.75, 95%BCa CI = 2.67–64.57) and medium-SEC (OR = 5.08, 95%BCa CI = 1.55–24.84) compared with high-SEC schools. High-SES adolescents in low-SEC schools were at greater risk compared with those in medium-SEC (OR = 5.94, 95%BCa CI = 1.94–17.29) and high-SEC schools (OR = 4.99, 95%BCa CI = 1.71–13.14). A social gradient was observed in medium-SEC (OR_low/high_ = 2.79, 95%BCa CI = 1.22–7.41) and high-SEC (OR_low/medium_ = 6.86, 95%BCa CI = 1.06–5.22*10^6^) schools.

**Conclusions:**

Physical activity and lunch at and outside school help to understand these differences. Implications for obesity prevention initiatives are discussed.

**Supplementary Information:**

The online version contains supplementary material available at 10.1186/s12889-021-11752-2.

## Background

Obesity during adolescence is a stronger predictor of obesity during adulthood than obesity in earlier ages of life [1, 2]. Identifying adolescent obesity clusters is essential in promoting prevention initiatives, to prevent its spread through social contagion and thus reduce its short- and long-term burden [3]. Most paediatric obesity is said to result from the interaction between susceptibility genes and unhealthy lifestyle habits such as poor nutrition, high sedentary behaviour, and insufficient physical activity (PA) [4–6].

These habits are in part shaped by the provisions and types of facilities available in peoples’ living environments, for example the presence of fast-food restaurants, parks, cycling paths and sport facilities [7–9]. These environmental factors can cause overweight and obesity prevalence disparities within the same country, and even within the same local area when the area contains a variety of direct life environments [10].

Disadvantaged areas are specifically subject to higher prevalence of overweight and obesity due to an unfavourable environment for adopting a healthy diet and a physically active lifestyle [11–13]. Among adolescents, those living in a neighbourhood and attending a school with lower socioeconomic composition (SEC) are at greater risk of overweight and obesity [10, 14, 15]. Neighbourhood SEC was also found to be more influential than household socioeconomic status (SES) [10].

Meanwhile, in the same local environment, adolescents can develop different PA and nutritional habits and thus have different body mass index (BMI) trajectories. It is acknowledged that those with a lower household SES, in a similar local environment, will be more likely to be overweight or obese than those with a higher household SES [16]. However, as shown by one study, this household SES effect may depend on the SES of these adolescents’ living environment [17]. Kim et al. (2020) showed that high-SES neighbourhoods were protective against obesity among higher-SES adolescents, but not among those with a low SES. Additionally, in low- and medium-SES neighbourhoods, obesity prevalence was similar between low- and high-SES adolescents. To our knowledge, this is the first study that has pointed out an interaction effect between the neighbourhood and the individual SES levels on adolescent obesity risk.

Between the school SEC and the student SES, such an interaction effect has not yet been reported. Often, researchers have employed multilevel models to control the environmental SES while investigating student SES and other independent variables without reporting potential interaction effects [10, 14, 15, 18]. For example, in the longitudinal study by Niu et al. (2019), a three-level hierarchical linear model was used to estimate children’s BMI growth trajectories within schools [14]. Results showed an inverse relationship between BMI and SES. However, this study did not indicate whether this relationship was specific to a particular school SEC or valid across all schools regardless of their SEC.

This information would be crucial to initiate obesity prevention programs and monitor with greater attention overweight and obesity trends among relevant schools and student subgroups. The lack of a social gradient in low-SEC schools would indicate the need to develop obesity prevention directed toward all adolescents, regardless of their SES. In higher-SEC schools, a social gradient would suggest the need for programs targeted primarily toward adolescents with a lower SES.

Given the role of nutrition and PA, two socially determined factors, on overweight and obesity, there is also a need to control these variables while investigating this relationship [4, 6, 19, 20]. Further, analysis of these variables by school SEC and student SES would indicate whether they significantly differ within schools according to student SES, or between schools within the same student-SES category. This information would help to determine whether social differences in overweight and obesity are due, in part, to different lifestyles, or to other environmental aspects.

The main objective of this study was to investigate the interaction effect of school SEC and adolescent SES, while controlling for PA, school lunch and household composition in the prevalence of overweight and obesity among a representative sample of French adolescents of the third most populous *département* of France. To avoid estimate biases due to BMI disparities between French territories, this study was conducted in a restricted territory (Bouches-du-Rhône, in the South-East of France, the third most populous *département*) characterized by lower levels of BMI [21], high poverty rates, social inequalities and segregation, and where more than 95% of the general adolescent population are enrolled in a high school [22, 23]. These four last territorial specificities are particularly relevant to estimating BMI differences according to school SEC and adolescent SES among a representative sample.

## Methods

### Context

Eighteen public high schools were randomly selected to reflect population geographical and socioeconomic disparities in this area according to the last census report in 2017 [22].

### Consent procedures

We asked the Rector of the Education Authority for administrative approval for inclusion. Eleven high schools in seven towns were finally included and approved in a written consent on March 19, 2019.

The study was also approved by the Aix-Marseille University Ethics Committee (No. 2019-23-05-003).

A parental consent form was distributed to students between March 25 and March 29, 2019, by their Physical Education (PE) teachers. They had 2 weeks to return the form.

### Participants

Of 1257 adolescents, 1149 (91.4%) returned an accepted parental consent form and were included in the study.

Data collection was conducted from April 3 to May 15, 2019, in classrooms under the supervision of a researcher and a PE teacher.

**Fig. 1:**
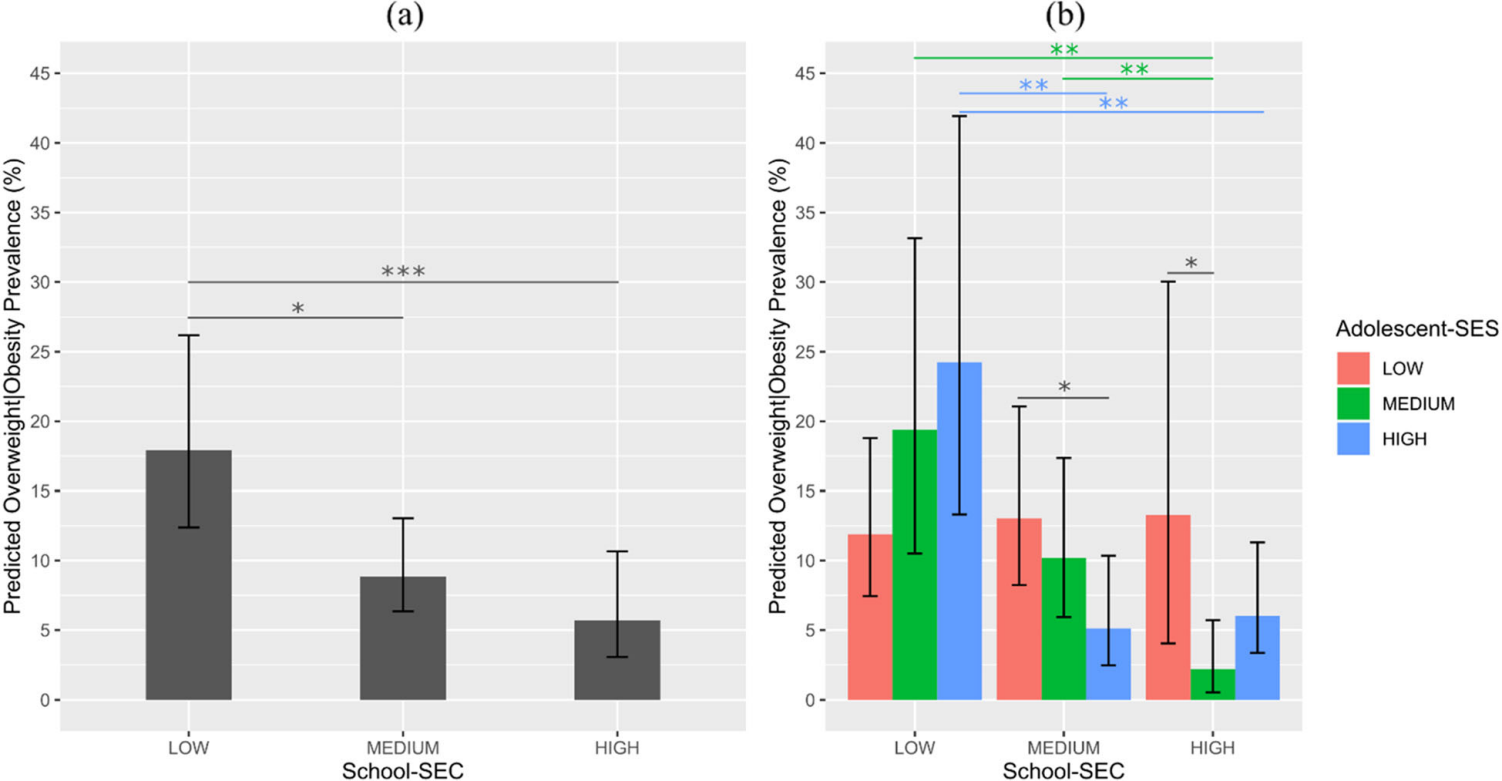
Overweight and obesity predicted prevalence from the multivariable model with interaction term according to (**a**) school SEC and (**b**) school SEC x adolescent SES**.** Note. *p-value:0.05, **p-value:0.01, ***p-value:0.001

Questionnaires with more than 50% of missing responses, aberrant or incoherent answers were excluded. Retained questionnaires with missing responses were substituted using multiple imputation with 50 replacement datasets using the R package *mice* (v. 3.6.2). The final sample size was 1038. Figure 1 in Additional File 1 details participant selection and exclusion.

### Measures

A specific questionnaire was developed for this study and is available in Additional File 2.

### BMI calculation and categorization

The adolescents self-reported their age, sex, height (cm) and weight (kg). BMI was calculated by dividing weight (kg) by height squared (cm^2^).

Six sets of adolescent outcomes according to age and sex were computed using the 2000 US Centers for Disease Control and Prevention Growth Charts: BMI Z-score, underweight (BMI < =5th percentile), normal weight (5th percentile <BMI <85th percentile), overweight (85th percentile <=BMI <95th percentile), obesity (BMI ≥95th percentile) and overweight or obesity (overweight|obesity: BMI ≥85th percentile) [24].

### Household composition

#### Family composition

The adolescents reported their family composition (whether they lived with both parents, only their father, only their mother, if they switched from time to time or whether they lived without parents). Family composition was divided into two categories (living with both/not living with both parents).

#### Number of siblings

The adolescents reported how many siblings they had and if they lived with them at home.

### Adolescent SES

The adolescents reported their father’s and mother’s occupation according to other French studies [25, 26]. Father’s and mother’s occupation were divided into two SES categories (low/high) based on the National Institute of Statistics and Economic Studies (INSEE), which provides average annual income by occupation and territory [22]. Father’s and mother’s SES were then combined into three categories (low/medium/high) to determine adolescent SES [25]. Adolescents with two low-SES parents were classified as low-SES, those with one low-SES and one high-SES parent as medium-SES and those with two high-SES parents as high-SES. When one of the parents’ occupations was missing, adolescent SES was based on the available occupation (father or mother). When both mother’s and father’s occupation were missing, adolescent SES was *NA*. All combination and transformed values are available in Additional File 1 Table 1.

Adolescents who did not live with their parents and who did not complete their parents’ occupations were excluded from the sample (*n* = 9, 0.8%).

### School SEC

High schools were classified into three SEC groups (low/medium/high) according to the proportions of low-, medium- and high-SES adolescents they included, and in order to have the most equal sample sizes for low-, medium- and high-SEC subgroups. Each adolescent was placed into one of the three groups according to their high school.

### Diet

#### School lunch

To control for diet, the adolescents reported their school meal status. Full board includes lunch and dinner at school. Half board includes lunch at school. No board includes no meal at school. They did not report specifically the numbers of days they ate at school. For readers’ information, usually, when an adolescent subscribes to a half-board program, he eats lunch 5 days (Monday to Friday) at school as the half-board payment includes these 5 days. Similarly, full-board pupils have their meals every day at school. Adolescents with no board almost never eat at school as there is no opportunity for day-to-day lunch payment. Students were divided into two groups (lunch at school/lunch outside school). In France, school lunch respects a strict balanced national norm diet (regardless of their SEC) adapted to adolescents’ needs [27]. This is thus a relevant, easily measurable indicator that reflects their lunch type and that can be used by public policies to shape adolescents’ diet habits.

### PA and its related metabolic equivalent task (MET)

#### Active transportation

The adolescents reported how many days they used their bicycle for at least 10 min consecutively and for how many hours per week (transformed into minutes/week). The energy expenditure for bike transportation was defined as 4 MET [28]. The adolescents’ active transportation weekly MET was calculated. This question was copied from the Global Physical Activity Questionnaire (https://www.who.int/‌ncds/‌surveillance/‌steps/‌GPAQ_FR.pdf) [29].

#### Sport participation

The adolescents reported how many days and hours they played sport outside of mandatory PE classes per week and indicated their main sport activity. These questions were copied from other sport questionnaires [26, 30]. Sport activity MET was calculated according to the updated compendium of Ainsworth et al. (2011) [31]. Unclassified sports were classified by a committee of four sports scientists and PE teachers according to the closest sport of the compendium in terms of energy expenditure. Individuals’ sport participation weekly MET was calculated. All details of MET by sports are available in Additional File 1 Table 2.

Additional questions were asked about their main sport activity (context of participation, years of experience, level of competition and amount of participation) but were not treated in the present study.

#### PA proxy measure

A total MET/week was calculated by multiplying active transportation and sport weekly MET. We then divided MET/week into three categories (low/medium/high) according to three relevant thresholds for long-term cardiovascular diseases risks [32]: low (< 600 MET × minutes per week or < 150 min per week of moderate intensity PA), moderate (600–3000 MET × minutes per week or 150–750 min per week) and high PA (> 3000 MET × minutes per week or > 750 min per week).

### Analysis

All statistical analyses were performed using R version 3.6.2.

First, sample description was performed for the whole sample and by school SEC.

Second, school lunch and PA level relationship with adolescent SES and school SEC was analysed. Cross-tables with chi-square statistics and multivariable logistic regression were used to determine whether they were significantly linked. The significance level was set at 0.05.

Third, overweight and obesity risk was analysed in univariable and multivariable logistic regression analyses with and without interaction term between adolescent SES and school SEC. An additional model using the forward and backward stepwise technique was performed. The goal was to create the simplest regression model that best fitted the study data. Model fit was compared using the Akaike Information Criterion (AIC), the Area Under Curve (AUC), and the specificity and sensitivity results. The best model was chosen according to these latter parameters assuming that (i) the lower the AIC, the better the model, (ii) the higher the AUC the better the model, (iii) and the higher the specificity and sensitivity, the better the model. For each model, the bootstrap method was used to account for generalizability error, and bias-corrected 95% confidence intervals were calculated based on 3000 resamples of the data (minimum recommended resamples: 1000) [33].

Fourth, the best model overweight and obesity predicted prevalence output was plotted either (i) separately by adolescent SES and school SEC if the interaction model fit the data less well or (ii) according to adolescent SES and school SEC if the interaction model fit the data better. Predicted prevalences were calculated using a marginal probability prediction with the package *emmeans* (v. 1.5.3). We also presented observed number and prevalence of overweight|obesity by school SEC and adolescent SES.

Finally, contrast analyses were performed using odds-ratio to compare overweight and obesity risk by variable category. Contrast analyses aimed to compare this risk within school SEC according to adolescent SES and between school SEC for each adolescent-SES category. These analyses help in determining whether a social gradient exists within all school SECs or is valid for only one or two specific school SECs. It also helps to determine whether school SEC is positively associated with a lower overweight and obesity risk among all adolescent-SES categories, in only one or two specific SES categories, or is not associated.

## Results

Table 1 provides overall sample descriptive statistics. Results showed a BMI Z-score below the international mean (Mean = − 0.09 ± 1.01). The sample comprised 6.3% underweight, 81.5% normal weight, 8.9% overweight and 3.4% obese adolescents. Overweight|obesity prevalence in this area was below national prevalence among adolescents aged 13–15 (18.0% vs. 12.2%).

**Table 1:** Descriptive Statistics across the whole sample and by school SEC

Variables	All		School SEC	
		Low (***n*** = 3)	Medium (***n*** = 5)	High (***n*** = 3)
**Age** (M, SD)	15.92 (1.33)	16.61 (1.16)	15.37 (1.24)	15.82 (1.26)
**Sex** (n, %)				
Boys	515 (49.6%)	186 (53.6%)	195 (51.6%)	142 (45.4%)
Girls	523 (50.4%)	161 (46.4%)	183 (48.4%)	171 (54.6%)
**Body Mass Index**				
Weight / Height (M, SD)	20.83 (3.32)	21.97 (3.8)	20.59 (3.09)	19.85 (2.57)
CDC Z-Scores (M, SD)	−0.09 (1.01)	0.11 (1.05)	−0.04 (0.97)	−0.37 (0.94)
Underweight	65 (6.3%)	16 (4.6%)	22 (5.8%)	27 (8.6%)
Normal Weight	846 (81.5%)	274 (79.0%)	308 (81.5%)	264 (84.3%)
Overweight	92 (8.9%)	38 (11.0%)	36 (9.5%)	18 (5.8%)
Obesity	35 (3.4%)	19 (5.5%)	12 (3.2%)	4 (1.3%)
Overweight|Obesity	127 (12.2%)	57 (16.4%)	48 (12.7%)	22 (7.0%)
**Physical Activity**				
Low	282 (27.2%)	142 (40.9%)	79 (20.9%)	61 (19.5%)
Medium	623 (60.0%)	162 (46.7%)	245 (64.8%)	216 (69.0%)
High	133 (12.8%)	43 (12.4%)	54 (14.3%)	36 (11.5%)
**Adolescent SES**				
Low	351 (33.8%)	214 (61.7%)	108 (28.6%)	29 (9.3%)
Medium	277 (26.7%)	63 (18.2%)	118 (31.2%)	96 (30.7%)
High	368 (35.5%)	50 (14.4%)	137 (36.2%)	181 (57.8%)
NA	42 (4.1%)	20 (5.8%)	15 (4.0%)	7 (2.2%)
**School Lunch** (n, %)				
Lunch outside school	490 (47.2%)	304 (87.6%)	189 (50.0%)	55 (17.6%)
Lunch at school	548 (52.8%)	43 (12.4%)	189 (50.0%)	258 (82.4%)
**Household Composition**				
Live with both parents	680 (65.5%)	218 (62.8%)	231 (61.1%)	231 (73.8%)
Do not live with both parents	358 (34.5%)	129 (37.2%)	147 (38.9%)	82 (26.2%)
Number of Siblings (M, SD)	2.13 (1.55)	2.9 (1.76)	1.92 (1.45)	1.54 (0.98)
**Overall**	1038 (100.0%)	347 (100.0%)	378 (100.0%)	313 (100.0%)

### School lunch and physical activity levels by school SEC

Adolescents enrolled in medium- and high-SEC schools were more likely to eat at school compared with those enrolled in low-SEC schools (OR_medium/low_ = 3.77, 95% CI = 2.44–5.90 and OR_high/low_ = 17.23, 95% CI = 10.66–28.43, respectively). Detailed analysis of school differences in lunch type are provided in Additional File 1 Tables 3 (cross-tables and chi-square statistics), 4 (multivariable logistic regression analyses) and 5 (interaction term contrast analysis).

Adolescents in medium- and high-SEC schools were more likely to have medium or high PA levels rather than low PA levels compared with those enrolled in low-SEC schools (OR_medium/low_ = 2.93, 95% CI = 1.93–4.50 and OR_high/low_ = 2.63, 95% CI = 1.61–4.32, respectively). Detailed analyses of school differences in PA levels are provided in Additional File 1 Tables 6 (cross-tables and chi-square statistics), 7 (cross-tables and chi-square statistics), 8 (multivariable logistic regression analyses) and 9 (interaction term contrast analysis).

Those who ate their lunch at school were not more likely to have higher levels of PA than those who ate their lunch outside school (OR = 0.77, 95% CI = 0.53–1.12).

### Overweight|obesity risk

Table 2 shows results of univariable and multivariable models without or with interaction term between adolescent SES and school SEC.

**Table 2:** Results from the univariable, full model and stepwise procedure with bias-corrected and accelerated-bootstrap confidence interval (BCa) in overweight|obesity

Variables	Univariable Analysis	Full Model without interaction with BCa	Full Model with interaction and BCa	Stepwise Model with BCa
Age	0.89 [0.77;1.02]	0.80** [0.68;0.95]	0.81* [0.70;0.97]	0.81* [0.69;0.98]
Sex				
Girls (ref.)				
Boys	1.29 [0.89;1.87]	1.53* [1.03;2.29]	1.54* [1.01;2.34]	1.53 [1.00;2.35]
Adolescent SES				
Low (ref.)				
Medium	0.76 [0.47;1.21]	0.97 [0.58;1.62]	1.78 [0.83;3.91]	1.78 [0.71;3.51]
High	0.59* [0.37;0.93]	0.94 [0.55;1.59]	2.37 [1.00;5.52]	2.38 [0.99;5.54]
School SEC				
Low (ref.)				
Medium	0.74 [0.49;1.12]	0.58 [0.33;1.01]	1.11 [0.54;2.32]	1.15 [0.56;2.28]
High	0.38*** [0.22;0.64]	0.34** [0.17;0.68]	1.14 [0.31;3.52]	1.24 [0.27;3.29]
School Lunch				
Outside of school (ref.)				
At school	0.70 [0.47;1.01]	1.08 [0.66;1.76]	1.16 [0.69;1.91]	–
Physical Activity				
Low PA (ref.)				
Medium PA	0.82 [0.55;1.24]	1.01 [0.65;1.6]	1.03 [0.65;1.65]	1.02 [0.64;1.65]
High PA	0.48* [0.22;0.95]	0.44* [0.19;0.93]	0.42* [0.18;0.95]	0.41* [0.17;0.90]
Household Composition				
Live with both parents (ref.)				
Do not live with both parents	1.13 [0.77;1.66]	1.01 [0.66;1.52]	1.01 [0.65;1.58]	–
Number of Siblings (M, SD)	1.14* [1.02;1.27]	1.10 [0.97;1.25]	1.12 [0.98;1.28]	1.11 [0.97;1.26]
Adolescent SES x School SEC				
Low Adolescent SES x Low School SEC			ref.	ref.
Medium Adolescent SES x Medium School SEC			0.43 [0.15;1.30]	0.43 [0.15;1.36]
High Adolescent SES x Medium School SEC			0.15** [0.05;0.56]	0.16** [0.04;0.50]
Medium Adolescent SES x High-School SEC			0.08* [0.00;0.62]	0.08* [0.00;0.70]
High Adolescent SES x High-School SEC			0.18* [0.04;0.89]	0.18* [0.04;0.82]
Aikake Information Criterion		729	718.52	714.9
Sensitivity		73.6%	79.3%	79.3%
Specificity		50.9%	50.1%	50.9%
Area Under Curve		0.653	0.691	0.690

*Note*. Outcome variable: overweight or obesity; Ref.: Reference category; *p-value:0.05, **p-value:0.01, ***p-value:0.001

### Models comparison

Comparison of the multivariable models without and with interaction terms showed a significant interaction effect between adolescent SES and school SEC. The interaction model fits the data better compared with the multivariable model without interaction term (AIC = 718.5 and AUC = 0.691 with a sensitivity of 79.3% and a specificity of 50.1% versus AIC = 729 and AUC = 0.653 with a sensitivity of 73.6% and a specificity of 50.9%). Hence, results from the multivariable model with interaction are presented below.

### Main effects

We observed an effect of age, sex, and PA level in overweight|obesity risk. Older adolescents and girls were less likely to be overweight|obese compared with younger adolescents and boys (OR = 0.81, 95% BCa CI = 0.70–0.97 and OR = 0.65, 95% BCa CI = 0.43–0.99, respectively). Similarly to univariable analysis, adolescents with a high PA level were less likely to be overweight|obese compared with those with a low PA level (OR = 0.42, 95% BCa CI = 0.18–0.95).

### Interaction effects analysis

Table 3 shows contrast analysis results from the multivariable model with interaction term within school SEC and by adolescent SES and between school SECs according to adolescent SES. Results are expressed as odds-ratio.

**Table 3:** Odds-Ratio Contrast Analysis of School SEC and Adolescent SES with bias-corrected and accelerated-bootstrap confidence intervals

**Within School**	**Adolescent-SES Contrasts**	**Odds-Ratio**
Low-SEC	Low-Medium	0.56 [0.26;1.21]
	Medium-High	0.75 [0.27;1.92]
	Low-High	0.42 [0.19;1.04]
Medium-SEC	Low-Medium	1.32 [0.63;2.84]
	Medium-High	2.11 [0.85;5.29]
	Low-High	2.79* [1.22;7.41]
High-SEC	Low-Medium	6.86* [1.06;5.22*10^6^]
	Medium-High	0.35 [0.00;1.63]
	Low-High	2.39 [0.55;7.85]
**Within SES**	**School SEC Contrasts**	**Odds-Ratio**
Low SES	Low-Medium	0.90 [0.44;1.79]
	Medium-High	0.98 [0.33;3.99]
	Low-High	0.88 [0.29;3.55]
Medium SES	Low-Medium	2.12 [0.80;5.47]
	Medium-High	5.08** [1.55;24.84]
	Low-High	10.75** [2.67;64.57]
High SES	Low-Medium	5.94** [1.94;17.29]
	Medium-High	0.84 [0.32;2.20]
	Low-High	4.99** [1.71;13.14]

Note. *p-value:0.05, **p-value:0.01, ***p-value:0.001

Within school SEC, a statistically significant social gradient was observed in medium- and high-SEC schools while no statistically significant difference was observed in low-SEC schools. In medium-SEC schools, low-SES adolescents were more likely to be overweight|obese compared with high-SES adolescents (OR = 2.79, 95% BCa CI = 1.22–7.41). In high-SES schools, low-SES adolescents were at greater risk compared with medium-SES adolescents (OR = 6.86, 95% BCa CI = 1.06–5.22*10^6^). In low-SEC schools, no statistically significant difference was observed but the predicted and observed prevalence (Table 4) appeared to be higher among high- (23.4%) and medium- (18.6%) compared with low-SES (11.4%) adolescents.

**Table 4:** Observed and predicted number and prevalence of overweight and obesity by adolescent-SES and school-SEC

Variables		School SEC					
		Low		Medium		High	
		Observed	Predicted	Observed	Predicted	Observed	Predicted
Adolescent SES	Low	29 (13.6%)	24 (11.4%)	19 (17.6%)	14 (12.9%)	5 (17.2%)	4 (13.7%)
	Medium	13 (20.6%)	12 (18.6%)	17 (14.4%)	12 (10.2%)	3 (3.1%)	2 (2.3%)
	High	12 (24.0%)	12 (23.4%)	9 (6.6%)	7 (5.2%)	14 (7.7%)	11 (6.3%)

Within adolescent SES, an absence of school-SEC effect in overweight|obesity risk for low-SES adolescents was observed. By contrast, medium-SES adolescents were at greater risk in low- and medium- compared with high-SEC schools (OR_low/high_ = 10.75, 95% BCa CI = 2.67–64.57 and OR_medium/high_ = 5.08, 95% BCa CI = 1.55–24.84, respectively). High-SES adolescents were at greater risk in low- compared with medium- and high-SEC schools (OR_low/medium_ = 5.94, 95% BCa CI = 1.94–17.29 and OR_low/high_ = 4.99, 95% BCa CI = 1.71–13.14).

Figure 1 shows the predicted prevalence from the multivariable model with interaction term with accelerated-bootstrap confidence interval for (a) main effects of school SEC and (b) interaction effects between adolescent SES and school SEC.

All other statistically significant differences (between adolescent SES and school SEC) are available in Additional File 1 Table 10. All statistically significant differences indicated an increased risk for adolescents with a lower SES and in lower-SEC schools.

## Discussion

The main objective of this study was to investigate the interaction effect of school SEC and adolescent SES in the prevalence of overweight and obesity among a representative sample of French adolescents of the third most populous *département* of France.

### Consistency of results with previous findings

In France, multiple studies have shown that overweight and obesity prevalence among the general population (children, adolescents and adults) varied according to the territory (*département)* 21. It is acknowledged that overweight and obesity prevalence is lower in the South-East of France – which is the area concerned by our study – compared with the national level.

Among adolescents, to our knowledge, no study has documented systematic territorial inequalities in overweight and obesity prevalence. A recent national study (2019) conducted among adolescents aged 13–15 showed an overweight|obesity prevalence of 18%, and an obesity prevalence of 5.2% [25]. In the present study, an overweight|obesity prevalence of 12.2% and an obesity prevalence of 3.4% were observed among adolescent students aged 13–19 from South-East France, which is consistent with overweight and obesity territory disparities observed among the general French population.

The previous national study also observed that lower-SES adolescents – measured by their parents’ occupations – were less likely to eat their lunch at school [25]. This result was also observed in the present study, but it was mainly explained by the school SEC instead of the adolescent SES. In each school SEC, the proportions of adolescents having their lunch at school was not different according to their SES. Meanwhile, school SEC was strongly linked to having lunch at school, ranging from 12.4% in low-SEC schools, to 50.0 and 82.4% in medium- and high-SEC schools respectively (Additional File 1, Tables 3–5).

Consistent with previous findings, an effect of PA was also observed in multivariable models, meaning that a high level of PA is negatively linked to overweight and obesity, regardless of adolescent SES or school SEC [13, 19]. Moreover, statistically significant social differences were also observed in overweight and obesity, as pointed out by previous national reports and international studies [10, 14, 15, 25].

### Novel findings of the study

The originality of this study was to investigate and analyse a potential interaction effect between school SEC and adolescent SES, which has not been documented before. Results from the multivariable model including lunch type, PA, sex, age and household composition, showed a statistically significant interaction effect between school SEC and adolescent SES.

The model with interaction effect fits the data better compared with the model without interaction.

We observed that low-SES adolescents had a similarly high overweight|obesity prevalence in all school SECs. By contrast, medium- and high-SES adolescents were at greater risk in low-SEC schools compared with higher-SEC schools. In addition, in low-SEC schools, no statistically significant difference across adolescent SES was observed but overweight|obesity prevalence appeared to be much higher among medium- and high-SES adolescents, while in medium-SEC and high-SEC schools, adolescent SES was statistically significantly negatively associated with overweight|obesity risk.

One might have supposed, in view of the strong correlation between lunch type and PA with school SEC and adolescent SES (see Additional File 1, Tables 3 to 8), that social differences in overweight and obesity could have been mainly due to these different life habits. However, observing these social differences after having adjusted the models for these confounders underlines the potential role of other factor(s) responsible for these differences.

In this study, the lunch type of the adolescents was controlled because in each school SEC the lunch respects a recommended national norm of balanced diet adapted to adolescents’ needs [27]. Our results could suggest then that what adolescents eat outside lunch might have a greater influence to explain their weight status and the subsequent social differences.

In low-SEC schools, an inversed social gradient was observed, with the highest overweight|obesity prevalence in this study ranging from 11.4% for low-SES adolescents to 18.6% for medium-SES and 23.4% for high-SES adolescents. In these schools, adolescents were less prone to eat at school, regardless of their SES.

Notably, a previous study has shown that lunch outside school is influenced by the surrounding restaurant offers [27]. It has been shown that fast-food restaurant proximity to schools is positively linked to adolescent BMI [7, 8]. Further, low-SEC school neighbourhoods are characterized by a higher density of fast-food restaurants [34, 35]. It is reasonable to think that adolescents from these schools mainly eat their lunch in fast-food restaurants, which could be an explanation of the high prevalences observed.

At the same time, previous studies have generally argued that the dietary habits of low-SES families were constituted by poor nutrients and increased snacking time to a greater extent than for higher-SES adolescents, both factors associated with an increase in BMI [11, 12, 34, 35]. However, the specific result of the present study in low-SEC schools could suggests that medium- and high-SES adolescents enrolled in these schools might also be particularly concerned by a bad diet outside of the lunch at school, which goes against traditional assumptions regarding the general higher-SES adolescent population.

These results should foster the development of studies to understand more clearly this phenomenon of social gradient inversion that, to our knowledge, has never been documented before. More generally, it should foster public policies to develop prevention programs toward all adolescents in low-SEC schools.

In medium- and high-SEC schools, the results show the presence of a more “conventional” social gradient where higher-SES adolescents were less at risk compared with low-SES adolescents [10, 14, 15]. In view of the existing literature, this result could be understood as reflecting both (i) the role of the surrounding restaurants offers that are of better quality [34, 35] and are less frequented by adolescents due to the large proportions who eat at school (82.4%), and (ii) the better dietary habits of higher-SES adolescents outside schools compared with low-SES adolescents. These interpretations are in line with most previous findings in the field.

In summary, these results show that the frequentation of higher-SEC school environments does not benefit all adolescents equally, depending on their SES. On the other hand, the unfavourable environment of low-SEC schools substantially affects all adolescents, regardless of their SES. This result has already been observed in academic performance, but has never previously been shown in overweight and obesity social studies [36, 37].

Further studies might try to understand how adolescents’ life habits are shaped in different school SECs. This could help to better understand the reality of under-represented groups in specific social contexts. The results might help to understand why (i) high-SES adolescents presented high overweight and obesity prevalence in low-SEC schools and (ii) low-SES adolescents still presented high overweight and obesity prevalence in medium- and high-SEC schools.

## Limitations

### Self-reported measures

#### Anthropometric measurements

Due to the very low number of medical doctors (MD) in schools in France (only 1000 MD for 12,000,000 students), and their overwork (only 57% of students had a health check in 2015), we chose to ask the adolescents to self-report their age, sex, height and weight [38]. However, this implies potential bias considering that some studies have shown discrepancies between self-reported and physical measures of height and weight. Principally, self-reported weight is underestimated by overweight and obese adolescents [39–41]. This led to an underestimation of overweight and obesity prevalence in self-report studies of overweight and obesity. In the present study, social differences in overweight and obesity might have been underestimated. In addition, studies showed that BMI accuracy based on self-report differed significantly between male and female, but not enough to entail incorrect weight status classification [41–43]. Finally, temporal changes in bias of BMI scores based on self-reported height and weight have declined, leading to more accurate BMI categorizations between 1988 and 2008. This has been explained by the changing social norms regarding overweight and obesity due to their increasing prevalence and acceptability among the population [44]. It suggests then that, in the present study, self-reported bias might be even more reduced given the rising trends of overweight and obesity. All these studies gave confidence regarding the results from this study based on self-reported height and weight for adolescent weight status classification. However, to our knowledge, no study has reported bias differences between SES. It would be interesting, in a further study, to perform sensitivity analysis to measure bias accurately in self-reported height and weight by sex and SES, ideally in a sample of French adolescents.

#### Physical activity

In this study, the adolescents self-reported their PA, including cycling and sports. Questions related to cycling were copied from the GPAQ questionnaire, which show moderate to good test-retest reliability in adult samples [45, 46]. A previous French study also showed that the GPAQ underestimated total PA compared with the accelerometer [45]. This discrepancy has been specified by a study by Cleland et al. (2014) which showed that highly active adults tended to over-report their total PA, while inactive adults tended to under-report their total sedentary time [47]. Another study showed that females tended to better report total moderate-to-vigorous PA (MVPA) compared to males [48]. Cleland et al. also found that BMI was not a predictor of discordance for total MVPA, while adults with higher level of education tended to report more precisely their total MVPA compared with those with lower level of education. More precisely, adults with lower level of education tended to over-report their total MVPA.

If all the above-mentioned discrepancies are similar between adults and adolescents – which has not been tested previously – it would imply that in the present study social differences in PA might have been underestimated, meaning that lower SES adolescents might have lower total PA than mentioned. Thus, the objective PA gap between high-SES and low-SES adolescents might be higher than measured in the present study. However, these interpretations might be tempered as, in the present study, we observed that school SEC was more linked to total PA than adolescent SES. However, previous studies that compare self-report measures of total PA with accelerometer did not control for the SEC of the living environment of participants. In addition, to our knowledge, no study has explicitly tested these discrepancies in a sample of adolescents.

Finally, concerning sport participation, specific question were added that were not included in the GPAQ. The adolescents were explicitly asked to report whether they played sport every week, the volume of sport participation, and the main sport activity practiced each week. In the current form of the GPAQ, questions related to sport are based on the perceived intensity of performed sports activity (low, medium and high) without asking explicitly what sports adolescents play. Consequently, the total estimated MET per week are computed by multiplying 3 (low intensity), 6 (moderate intensity) or 8 (high intensity) by the total volume of participation in low, moderate or vigorous PA. However, sports are various in their form and intensity, which has been measured with the total oxygen cost of each sport in laboratory or field experiments [31]. To overcome this bias, we asked the adolescents to report the sport they practice the most each week and then compute adolescent sport total/MET according to the Compendium of Ainsworth et al. [31] In our opinion, this provides potentially better reliability of sport total/MET computed from these questions in comparison with the sport questions used in the GPAQ. Fortunately, consistently with previous studies, it was observed that PA was negatively associated with overweight and obesity risk, which adds confidence regarding the reliability of questions used for public health assessment. Nevertheless, a further study should specifically compare self-reported PA (including specific questions about sports) to direct measurements on a sample of French adolescents. It would help to establish whether the results observed concerning PA were under- or over-estimated, and whether subgroups’ characteristics are predictors of self-reported PA discordance compared with direct measurements (e. g. accelerometer).

### Sample size

Finally, French high schools are characterized by strong social segregation [23]. The present study highlights this phenomenon, where high-SEC schools had low numbers of low-SES adolescents, and low-SEC schools had low numbers of high-SES adolescents. This observed disparity clearly lowered the power of the test when comparing small adolescent subgroups with bigger ones, which is observed in large confidence intervals (for example low-SES compared with high-SES adolescents in high-SEC schools). One could suggest the development of mixed methods to better understand the reality of under-represented groups in specific school SECs. Qualitative studies would help to understand why high-SES adolescents presented high overweight and obesity prevalence in low-SEC schools. Similarly, this could also be undertaken in high-SEC schools for low-SES adolescents. Measuring these crossed perceptions could help to understand adolescent development in different social settings, while having parent(s) stemming from the same social class.

## Conclusion

This is the first study that has examines the interaction effect between school SEC and adolescent SES in overweight and obesity risk among a representative sample of adolescents of the third most populous *département* of France. The results showed a significant interaction effect, presenting (i) low-SES adolescents, regardless of their high-school SEC, and (ii) low-SEC high schools, regardless of adolescent SES, as high priority targets for obesity prevention initiatives. In addition, a social gradient inversion has been observed in low-SEC schools where higher-SEC adolescents had higher prevalence of overweight and obesity compared with low-SES adolescents. These results may foster the development of obesity prevention initiatives directed toward specific schools and adolescent social subgroups, for example through incentives to have lunch at school and/or nutrition and PA educational programs.

## Supplementary Information

**Additional file 1 Supplementary Fig. 1.** Study Participants Selection and Filtering for Analysis. **Supplementary Table 1**. Father and Mother Occupations Combined and Translated to Three Adolescent-SES levels. **Supplementary Table 2**. List of sports activities (name, number, proportion) declared by adolescents and their related metabolic equivalent and mean of computed MET/min/week. **Supplementary Table 3**. School Lunch by School SEC and Adolescent SES. **Supplementary Table 4.** Results from the full model without and with interaction in School Lunch. **Supplementary Table 5**. School Lunch Odds-Ratio Contrast Analysis of School SEC and Adolescent SES. **Supplementary Table 6.** PA Level by School SEC and Adolescent SES. **Supplementary Table 7.** PA Level by School SEC and Adolescent SES. **Supplementary Table 8**. Results from the full model without and with interaction in Medium|High PA Level. **Supplementary Table 9.** PA Level Odds-Ratio Contrast Analysis of School-SEC and Adolescent SES. **Supplementary Table 10.** All significant crossed interaction effects. **Supplementary Table 11.** Results from the univariable, full model without and with interaction to estimate BMI Z-Scores.

**Additional file 2 **Questionnaire. Adolescent students' physical and sport activity.

## Data Availability

The datasets used and/or analysed during the current study are available from the corresponding author on reasonable request.

## Abbreviations

AIC: Akaike Information Criterion
AUC: Area Under Curve
BMI: Body Mass Index
MET: Metabolic Equivalent of Task
INSEE: National Institute of Statistics and Economic Studies
OR: Odds-Ratio
Overweight/obesity: Overweight or obesity
PA: Physical Activity
PE: Physical Education
SEC: Socioeconomic Composition
SES: Socioeconomic Status
95%BCa CI: 95% accelerated-bootstrap confidence interval
