# Supplementary material for: Overweight and obesity by school socioeconomic composition and adolescent socioeconomic status: a school-based study
Source: BMC Public Health. 2021 Oct 11;21:1837. doi: 10.1186/s12889-021-11752-2 (PMC8507235; doi:10.1186/s12889-021-11752-2)
Supplement: Supplementary file 1 — Additional file 1 Supplementary Fig. 1. Study Participants Selection and Filtering for Analysis. Supplementary Table 1. Father and Mother Occupations Combined and Translated to Three Adolescent-SES levels. Supplementary Table 2. List of sports activities (name, number, proportion) declared by adolescents and their related metabolic equivalent and mean of computed MET/min/week. Supplementary Table 3. School Lunch by School SEC and Adolescent SES. Supplementary Table 4. Results from the full model without and with interaction in School Lunch. Supplementary Table 5. School Lunch Odds-Ratio Contrast Analysis of School SEC and Adolescent SES. Supplementary Table 6. PA Level by School SEC and Adolescent SES. Supplementary Table 7. PA Level by School SEC and Adolescent SES. Supplementary Table 8. Results from the full model without and with interaction in Medium|High PA Level. Supplementary Table 9. PA Level Odds-Ratio Contrast Analysis of School-SEC and Adolescent SES. Supplementary Table 10. All significant crossed interaction effects. Supplementary Table 11. Results from the univariable, full model without and with interaction to estimate BMI Z-Scores. [file 12889_2021_11752_MOESM1_ESM.docx]

**Additional File 1.**

**
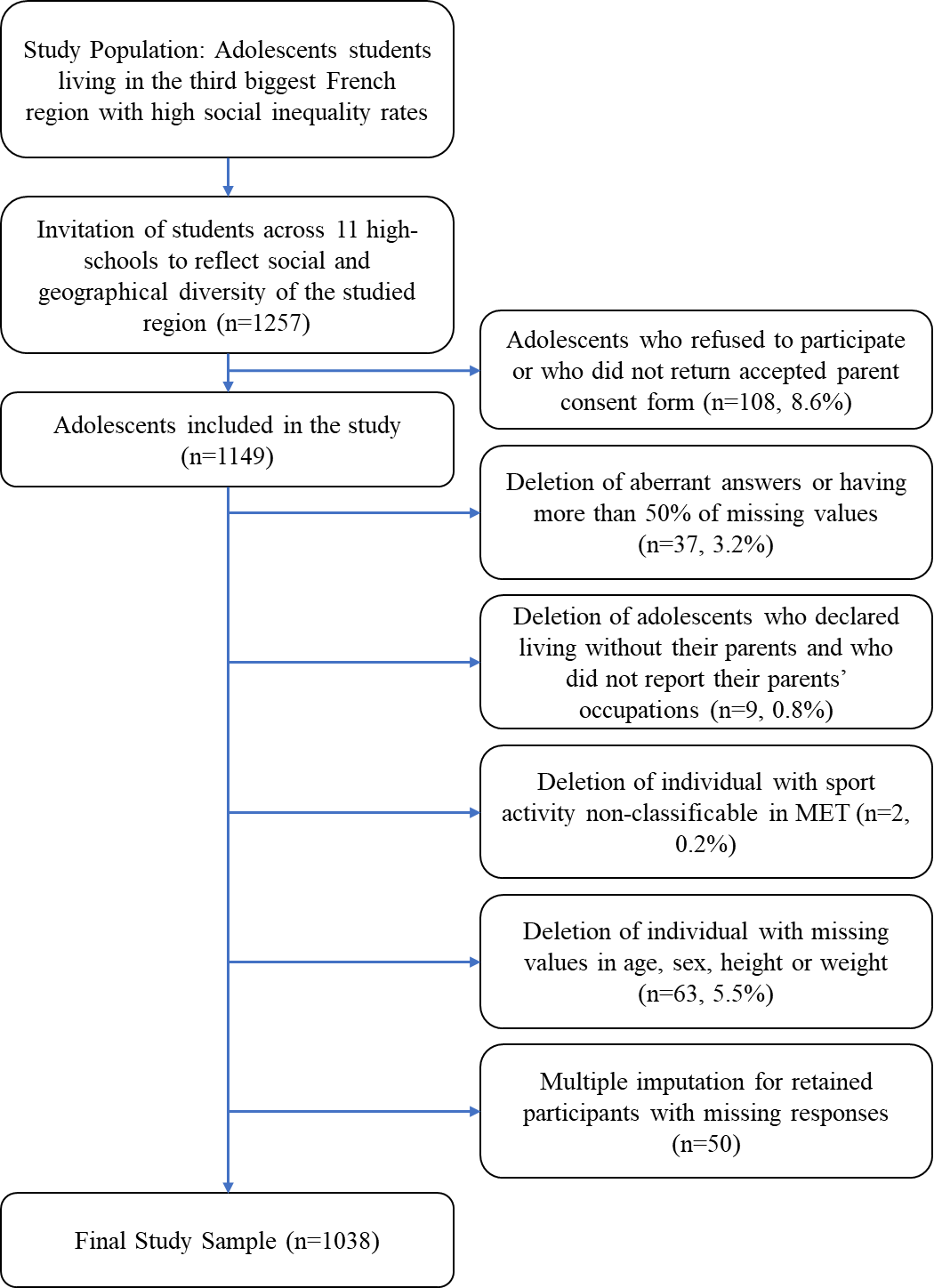
**

**Supplementary Figure 1.** Study Participants Selection and Filtering for Analysis

| **Supplementary Table 1. Father and Mother Occupations Combined and Translated to Three Adolescent-SES levels** | | | | | | | | | | | | | | |
| --- | --- | --- | --- | --- | --- | --- | --- | --- | --- | --- | --- | --- | --- | --- |
|  | Father | 1 | 2 | 3 | 4 | 5 | 6 | 7 | 8 | 9 | 10 | NA | Overall |  |
| Mother |  |  |  |  |  |  |  |  |  |  |  |  |  |  |
| 1 | | 0 (0·0%) | 1 (0·1%) | 0 (0·0%) | 0 (0·0%) | 0 (0·0%) | 0 (0·0%) | 1 (0·1%) | 0 (0·0%) | 0 (0·0%) | 0 (0·0%) | 0 (0·0%) | 2 (0·2%) |  |
| 2 | | 3 (0·3%) | 38 (3·7%) | 10 (1·0%) | 6 (0·6%) | 7 (0·7%) | 4 (0·4%) | 1 (0·1%) | 2 (0·2%) | 0 (0·0%) | 1 (0·1%) | 8 (0·8%) | 80 (7·7%) |  |
| 3 | | 2 (0·2%) | 28 (2·7%) | 81 (7·8%) | 20 (1·9%) | 9 (0·9%) | 3 (0·3%) | 3 (0·3%) | 11 (1·1%) | 0 (0·0%) | 1 (0·1%) | 10 (1·0%) | 168 (16·2%) |  |
| 4 | | 0 (0·0%) | 28 (2·7%) | 42 (4·0%) | 49 (4·7%) | 27 (2·6%) | 8 (0·8%) | 3 (0·3%) | 3 (0·3%) | 3 (0·3%) | 3 (0·3%) | 18 (1·7%) | 184 (17·7%) |  |
| 5 | | 1 (0·1%) | 34 (3·3%) | 36 (3·5%) | 14 (1·3%) | 71 (6·8%) | 26 (2·5%) | 8 (0·8%) | 12 (1·2%) | 2 (0·2%) | 3 (0·3%) | 30 (2·9%) | 237 (22·8%) |  |
| 6 | | 0 (0·0%) | 0 (0·0%) | 3 (0·3%) | 0 (0·0%) | 0 (0·0%) | 3 (0·3%) | 0 (0·0%) | 1 (0·1%) | 0 (0·0%) | 1 (0·1%) | 2 (0·2%) | 10 (1·0%) |  |
| 7 | | 0 (0·0%) | 2 (0·2%) | 1 (0·1%) | 0 (0·0%) | 0 (0·0%) | 0 (0·0%) | 1 (0·1%) | 0 (0·0%) | 0 (0·0%) | 0 (0·0%) | 2 (0·2%) | 6 (0·6%) |  |
| 8 | | 0 (0·0%) | 9 (0·9%) | 12 (1·2%) | 4 (0·4%) | 15 (1·4%) | 3 (0·3%) | 2 (0·2%) | 14 (1·3%) | 1 (0·1%) | 1 (0·1%) | 10 (1·0%) | 71 (6·8%) |  |
| 9 | | 3 (0·3%) | 37 (3·6%) | 28 (2·7%) | 7 (0·7%) | 34 (3·3%) | 24 (2·3%) | 2 (0·2%) | 16 (1·5%) | 8 (0·8%) | 11 (1·1%) | 30 (2·9%) | 200 (19·3%) |  |
| 10 | | 0 (0·0%) | 4 (0·4%) | 4 (0·4%) | 1 (0·1%) | 1 (0·1%) | 1 (0·1%) | 2 (0·2%) | 0 (0·0%) | 1 (0·1%) | 13 (1·3%) | 5 (0·5%) | 32 (3·1%) |  |
| NA | | 0 (0·0%) | 11 (1·1%) | 3 (0·3%) | 2 (0·2%) | 5 (0·5%) | 1 (0·1%) | 2 (0·2%) | 0 (0·0%) | 0 (0·0%) | 2 (0·2%) | 22 (2·1%) | 48 (4·6%) |  |
| Overall | | 9 (0·9%) | 192 (18·5%) | 220 (21·2%) | 103 (9·9%) | 169 (16·3%) | 73 (7·0%) | 25 (2·4%) | 59 (5·7%) | 15 (1·4%) | 36 (3·5%) | 137 (13·2%) | 1038 (100·0%) |  |
| **Note.** 1: Farmers; 2: Merchants; 3: Intellectual occupations (white collar workers, medical doctors, etc.); 4: Intermediate occupations (teachers, nurses, etc.); 5: Salaried employees; 6: Manual workers; 7: Retirees; 8: Unemployed; 9: At home/Unemployed; 10: Other. SES Correspondence: Low SES; Medium SES; High SES | | | | | | | | | | | | | | |

| **Supplementary Table 2. List of sports activities (name, number, proportion) declared by adolescents and their related metabolic equivalent and mean of computed MET/min/week** | | | | | |  |
| --- | --- | --- | --- | --- | --- | --- |
| Sport | Metabolic Equivalent Task | Estimated with | Weekly PA Levels * MET (MEAN (SD)) | n | Proportion | |
| AIKIDO | 6 | boxing bag, punching bag | 6150 (721) | 2 | 0·2% | |
| AMERICAN FOOTBALL | 9 | competitive football | 7470 (NA) | 1 | 0·1% | |
| ARCHERY | 3·5 | non competitive | 720 (170) | 2 | 0·2% | |
| ARTISTIC GYMNASTIC | 4 | gymnastics, general | 1872 (611) | 2 | 0·2% | |
| ATHLETICS | 10 | running on track, team practice | 4819 (2925) | 9 | 0·9% | |
| BADMINTON | 7 | badminton, competitive (Taylor Code 450) | 2700 (1052) | 14 | 1·3% | |
| BALLET DANCE | 4·8 | ballet or modem, twist, jazz, tap, jitterbug | 4690 (NA) | 1 | 0·1% | |
| BASKET-BALL | 8 | basketball, game (Taylor Code 490) | 4362 (3509) | 58 | 5·6% | |
| BIKING | 6 | bicycling, 10-11·9 mph, leisure, slow, light effort | 4942 (2817) | 10 | 1·0% | |
| BMX | 8·5 | bicycling, BMX or mountain | 3390 (NA) | 1 | 0·1% | |
| BODYBUILDING | 6 | weight lifting (free weight, nautilus or universal-type), power lifting or body building, vigorous effort (Taylor Code 210) | 2114 (1654) | 37 | 3·6% | |
| BOXING | 12 | boxing, in ring, general | 4598 (2698) | 46 | 4·4% | |
| BREAKDANCING | 4·8 | bicycling, BMX or mountain | 1038 (602) | 2 | 0·2% | |
| CHEERLEADING | 7 | aerobic, high-impact | 2160 (NA) | 1 | 0·1% | |
| CIRCUS | 4 | gymnastics, general; juggling | 1380 (NA) | 1 | 0·1% | |
| CLIMBING | 11 | rock climbing, ascending rock | 5675 (2671) | 16 | 1·5% | |
| COLLECTIVE SPORT | 8 | basketball, game (Taylor Code 490) | 2400 (NA) | 1 | 0·1% | |
| COMBAT SPORTS | 9 | boxing, sparring | 4320 (NA) | 1 | 0·1% | |
| CROSS-COUNTRY CYCLING | 10 | bicycling, 14-15·9 mph, racing or leisure, fast, vigorous effort | 3922 (2582) | 10 | 1·0% | |
| CROSSFIT | 8 | circuit training, including some aerobic movement with minimal rest, general | 1218 (704) | 2 | 0·2% | |
| DANCE RUN CLIMB | 10 | aerobic, step, with 10 - 12 inch step | 2520 (NA) | 1 | 0·1% | |
| DANCING | 4·8 | ballet or modem, twist, jazz, tap, jitterbug | 1881 (1087) | 55 | 5·3% | |
| DOWNHILL CYCLING | 8 | bicycling, general | 3480 (NA) | 1 | 0·1% | |
| FENCING | 6 | fencing | 4980 (NA) | 1 | 0·1% | |
| FITNESS | 5·5 | health club exercise, general (Taylor Code 160) | 2815 (1514) | 4 | 0·4% | |
| FREE DIVING | 5 | snorkeling (Taylor Code 320) | 2940 (NA) | 1 | 0·1% | |
| FRENCH BOXING | 12 | boxing, in ring, general | 2400 (NA) | 1 | 0·1% | |
| GOLF | 4·5 | Golf, walking and carrying clubs | 810 (NA) | 1 | 0·1% | |
| GYMNASTIC | 4 | gymnastics, general | 2224 (1283) | 18 | 1·7% | |
| HANDBALL | 12 | handball, general (Taylor Code 520) | 4057 (1916) | 15 | 1·4% | |
| HIKING | 6 | hiking, cross country (Taylor Code 040) | 289 (NA) | 1 | 0·1% | |
| HIP-HOP DANCE | 10 | aerobic, step, with 10 - 12 inch step | 3766 (2529) | 3 | 0·3% | |
| HORSE RIDING | 6·5 | horseback riding, trotting | 2236 (967) | 20 | 1·9% | |
| JUDO | 10 | judo, jujitsu, karate, kick boxing, tae kwan do | 6041 (3752) | 19 | 1·8% | |
| JUJITSU | 10 | judo, jujitsu, karate, kick boxing, tae kwan do | 10140 (NA) | 1 | 0·1% | |
| KARATE | 10 | judo, jujitsu, karate, kick boxing, tae kwan do | 2895 (1361) | 4 | 0·4% | |
| KARTING | 4 | motor-cross | 1824 (NA) | 1 | 0·1% | |
| KAYAK | 5 | kayaking | 1650 (NA) | 1 | 0·1% | |
| KICK-BOXING | 10 | judo, jujitsu, karate, kick boxing, tae kwan do | 1800 (NA) | 1 | 0·1% | |
| MIXED-MARTIAL ARTS | 10 | judo, jujitsu, karate, kick boxing, tae kwan do | 5209 (409) | 2 | 0·2% | |
| MODERN JAZZ DANCE | 4·8 | ballet or modem, twist, jazz, tap, jitterbug | 1416 (NA) | 1 | 0·1% | |
| MOTOCROSS RIDING | 4 | motor-cross | 3660 (3121) | 4 | 0·4% | |
| MOTORCYCLE SPORT | 4 | motor-cross | 2640 (NA) | 1 | 0·1% | |
| NON-SPORT-PLAYER | 1 | basic level | 605 (801) | 362 | 34·9% | |
| *NO-REPORTED* | *9·1 (Imputed)* | not applicable | 2727 (1458) | *11* | 1·1% | |
| PADDLE | 4 | paddle boat | 720 (NA) | 1 | 0·1% | |
| PAINTBALL | 6 | jog/walk combination | 2880 (NA) | 1 | 0·1% | |
| PARKOUR | 9 | running, cross country | 3291 (1565) | 4 | 0·4% | |
| PETANQUE (FRENCH BOWLS) | 2 | shooting sport | 1080 (NA) | 1 | 0·1% | |
| RESCUE | 10 | swimming, breaststroke, general | 3900 (NA) | 1 | 0·1% | |
| ROLLER HOCKEY | 8 | Hockey field | 1658 (NA) | 1 | 0·1% | |
| ROWING | 8·5 | rowing, stationary, 150 watts, vigorous effort | 9679 (2646) | 2 | 0·2% | |
| RUGBY FOOTBALL | 10 | rugby | 4471 (2331) | 14 | 1·3% | |
| RUNNING | 7 | jogging, general | 2714 (2335) | 17 | 1·6% | |
| SAILING | 3 | sailing, boat and board sailing, windsurfing, ice sailing, general | 1007 (279) | 2 | 0·2% | |
| SHOOTING SPORT | 2 | standing up | 396 (NA) | 1 | 0·1% | |
| SKATEBOARDING | 5 | skateboarding | 3570 (3153) | 5 | 0·5% | |
| SKIING | 8 | skiing, downhill, vigorous effort, racing | 9720 (11710) | 2 | 0·2% | |
| SNOWBOARDING | 7 | skiing, general | 3210 (976) | 2 | 0·2% | |
| SOCCER | 10 | soccer competitive | 4723 (2754) | 129 | 12·4% | |
| STEP | 8·5 | aerobic, step, with 6 - 8 inch step | 2010 (NA) | 1 | 0·1% | |
| STREET WORKOUT | 8 | calisthenics (e.g. pushups, situps, pullups, jumping jacks), heavy, vigorous effort | 3079 (737) | 2 | 0·2% | |
| STRENGHT TRAINING | 8 | calisthenics (e.g. pushups, situps, pullups, jumping jacks), heavy, vigorous effort | 2830 (1409) | 8 | 0·8% | |
| SURFING | 3 | surfing, body or board | 705 (106) | 2 | 0·2% | |
| SWIMMING | 8 | swimming, sidestroke, general | 2906 (2263) | 21 | 2·0% | |
| SYNCHRONIZED SWIMMING | 8 | swimming, synchronized | 3300 (NA) | 1 | 0·1% | |
| TABLE TENNIS | 4 | table tennis, ping pong (Taylor Code 410) | 2310 (330) | 4 | 0·4% | |
| TAEKWONDO | 10 | judo, jujitsu, karate, kick boxing, tae kwan do | 3509 (801) | 6 | 0·6% | |
| TAYVUADO | 10 | judo, jujitsu, karate, kick boxing, tae kwan do | 5520 (NA) | 1 | 0·1% | |
| TENNIS | 7 | tennis, general | 2664 (1535) | 30 | 2·9% | |
| THAÏ BOXING | 12 | boxing, in ring, general | 5647 (2803) | 5 | 0·5% | |
| TRAMPOLINING | 3·5 | trampoline | 1140 (NA) | 1 | 0·1% | |
| TRIATHLON | 10 | running, 6 mph (10 min/mile) | 6240 (NA) | 1 | 0·1% | |
| VOLLEYBALL | 8 | volleyball, competitive, in gymnasium | 4000 (1722) | 14 | 1·3% | |
| VTT ENDURO | 10 | bicycling, 14-15·9 mph, racing or leisure, fast, vigorous effort | 6000 (NA) | 1 | 0·1% | |
| WALKING | 5 | walking, 4·0 mph, level, firm surface, very brisk pace | 1135 (375) | 4 | 0·4% | |
| WALKING FAST | 8 | walking, 5·0 mph | 5110 (3577) | 2 | 0·2% | |
| WATERPOLO | 10 | water polo | 1680 (NA) | 1 | 0·1% | |
| WINDSURFING | 6 | sailing, boat and board sailing, windsurfing, ice sailing, general (Taylor Code 235) | 1759 (NA) | 1 | 0·1% | |
| YOSEIKAN BUDO | 10 | judo, jujitsu, karate, kick boxing, tae kwan do | 3540 (NA) | 1 | 0·1% | |
| ZUMBA DANCE | 6·5 | aerobic, general | 1897 (NA) | 1 | 0·1% | |
| All |  |  |  | 1038 | 100·0% | |

| **Supplementary Table 3. School Lunch by School SEC and Adolescent SES** | | | | | | | |  | |  | |  | |  | |
| --- | --- | --- | --- | --- | --- | --- | --- | --- | --- | --- | --- | --- | --- | --- | --- |
| Variables | | School SEC | | | | | | | | | | | | Chi-squared (p-value)^b^ | |
|  |  | Low | | | | Medium | | | | High | | | |  |  |
|  |  | School Lunch | | | | School Lunch | | | | School Lunch | | | |  |  |
|  |  | Outside of School | | At School | | Outside of School | | At School | | Outside of School | | At School | |  |  |
| ADOLESCENT SES | LOW | | 190 (88·8%) | | 24 (11·2%) | | 67 (62·0%) | | 41 (38·0%) | | 8 (27·6%) | | 21 (72·4%) | | 66·991*** (2·839e-15) |
|  | MEDIUM | | 54 (85·7%) | | 9 (14·3%) | | 53 (44·9%) | | 65 (55·1%) | | 16 (16·7%) | | 80 (83·3%) | | 73·481*** (< 2·2e-16) |
|  | HIGH | | 41 (82·0%) | | 9 (18·0%) | | 58 (42·3%) | | 79 (57·7%) | | 31 (17·1%) | | 150 (82·9%) | | 76·861*** (< 2·2e-16) |
|  | *NA* | | *19 (95·0%)* | | *1 (5·0%)* | | *11 (73·3%)* | | *4 (26·7%)* | | *0 (0·0%)* | | *7 (100·0%)* | | 22·972*** (1·027e-05) |
| Chi-squared (p-value)^a^ | | 1·8117 (0·4042) | | | | 10·568** (0·005073) | | | | 2·0167 (0·3648) | | | |  | |
| Overall | | 304 (87·6%) | | 43 (12·4%) | | 189 (50·0%) | | 189 (50·0%) | | 55 (17·6%) | | 258 (82·4%) | | 325·75***^c^ (< 2·2e-16) | |
| **Note.** ^a^The Chi-Squared have been computed within School SEC and without NA values for Adolescent SES. ^b^The Chi-Squared have been computed within Adolescent SES. ^c^Aggregated Chi-squared statistics between School SEC and School Lunch. **p-value<0·01; ***p-value<0·001 | | | | | | | | | | | | | | | |

| **Supplementary Table 4. Results from the full model without and with interaction in School Lunch** | | | |  |
| --- | --- | --- | --- | --- |
| Variables |  | Full Model without Interaction | Full Model with interaction | |
| Age |  | 0·74*** [0·65;0·84] | 0·73*** [0·64;0·83] | |
| Sex |  |  |  | |
|  | Girls (ref.) |  |  | |
|  | Boys | 0·82 [0·6;1·12] | 0·81 [0·59;1·11] | |
| Adolescent SES |  |  |  | |
|  | Low (ref.) |  |  | |
|  | Medium | 1·84** [1·22;2·78] | 1·20 [0·5;2·71] | |
|  | High | 2·00*** [1·34;2·98] | 1·65 [0·67;3·8] | |
| School SEC | |  |  | |
|  | Low (ref.) |  |  | |
|  | Medium | 3·77*** [2·44;5·9] | 2·93*** [1·59;5·49] | |
|  | High | 17·23*** [10·66;28·43] | 14·68*** [5·87;39·92] | |
| Physical Activity |  |  |  | |
|  | Low (ref.) |  |  | |
|  | Medium\|High | 0·79 [0·54;1·14] | 0·78 [0·54;1·14] | |
| Household Composition | |  |  | |
|  | Live with both parents (ref.) |  |  | |
|  | Do not live with both parents | 1·30 [0·93 ;1·81] | 0·69* [0·51 ;0·94] | |
|  | Number of Siblings (M, SD) | 0·81*** [0·72 ;0·91] | 1·05 [0·95 ;1·16] | |
| Adolescent SES x School SEC | |  |  | |
|  | Medium Adolescent SES x Medium School SEC |  | 1·74 [0·65 ;4·91] | |
|  | High Adolescent SES x Medium School SEC |  | 1·44 [0·54 ;4·10] | |
|  | Medium Adolescent SES x High School SEC |  | 1·78 [0·48 ;6·62] | |
|  | High Adolescent SES x High School SEC |  | 1·15 [0·32 ;4·09] | |
| AIC |  | 1009 | 1015 | |
| **Note.** Ref. : Reference category; *p-value<0·05; **p-value<0·01; ***p-value<0·001. AIC: Aikaike Information Criterion. | | | | |

| **Supplementary Table 5. School Lunch Odds-Ratio Contrast Analysis of School SEC and Adolescent SES** | | |
| --- | --- | --- |
| Within School SEC | Adolescent-SES Contrasts |  |
| Low-SEC Schools |  | Estimate Differences |
|  | Low-Medium | 0·83 [0·22;3·14] |
|  | Medium-High | 0·73 [0·14;3·75] |
|  | Low-High | 0·61 [0·16;2·37] |
| Medium-SEC Schools |  |  |
|  | Low-Medium | 0·48 [0·20;1·15] |
|  | Medium-High | 0·88 [0·39;2·00] |
|  | Low-High | 0·42* [0·18;0·99] |
| High-SEC Schools |  |  |
|  | Low-Medium | 0·47 [0·10;2·24] |
|  | Medium-High | 1·13 [0·39;3·29] |
|  | Low-High | 0·53 [0·12;2·25] |
| Within Adolescent SES | School-SEC Contrasts |  |
| Low-SES Adolescents |  | Estimate Differences |
|  | Low-Medium | 0·34** [0·13;0·91] |
|  | Medium-High | 0·20* [0·05;0·87] |
|  | Low-High | 0·07*** [0·02;0·31] |
| Medium-SES Adolescents |  |  |
|  | Low-Medium | 0·20** [0·05;0·73] |
|  | Medium-High | 0·19*** [0·07;0·57] |
|  | Low-High | 0·04*** [0·01;0·16] |
| High-SES Adolescents |  |  |
|  | Low-Medium | 0·24* [0·06;0·88] |
|  | Medium-High | 0·25*** [0·11;0·58] |
|  | Low-High | 0·06*** [0·02;0·23] |
| **Note.** *p-value<0·05; **p-value<0·01; ***p-value<0·001 | | |

| **Supplementary Table 6. PA Level by School SEC and Adolescent SES** | | | | | | | | | | | | | | | | | | | |
| --- | --- | --- | --- | --- | --- | --- | --- | --- | --- | --- | --- | --- | --- | --- | --- | --- | --- | --- | --- |
| Variables | | | School SEC | | | | | | | | | | | | | | | | Chi-squared (p-value)^b^ |
|  |  |  | LOW | | | | | MEDIUM | | | | | HIGH | | | | | |  |
|  |  |  | PA Level | | | | | PA Level | | | | | PA Level | | | | | |  |
|  |  |  | Low | | Medium | | High | Low | Medium | | High | | Low | | Medium | | High | |  |
| ADOLESCENT- SES | LOW | 92 (43·0%) | | 101 (47·2%) | | 21 (9·8%) | | 28 (25·9%) | | 65 (60·2%) | | 15 (13·9%) | | 8 (27·6%) | | 20 (69·0%) | | 1 (3·4%) | 12·863** (0·01197) |
|  | MEDIUM | 25 (39·7%) | | 30 (47·6%) | | 8 (12·7%) | | 25 (21·2%) | | 75 (63·6%) | | 18 (15·3%) | | 22 (22·9%) | | 68 (70·8%) | | 6 (6·3%) | 13·071* (0·01094) |
|  | HIGH | 17 (34·0%) | | 22 (44·0%) | | 11 (22·0%) | | 21 (15·3%) | | 96 (70·1%) | | 20 (14·6%) | | 31 (17·1%) | | 123 (68·0%) | | 27 (14·9%) | 12·879* (0·01188) |
|  | NA | 8 (40·0%) | | 9 (45·0%) | | 3 (15·0%) | | 5 (33·3%) | | 9 (60·0%) | | 1 (6·7%) | | 0 (0·0%) | | 5 (71·4%) | | 2 (28·6%) | 5·118 (0·2754) |
| Chi-squared (p-value)^a^ | | | 5·8663 (0·2094) | | | | | 4·4191 (0·3522) | | | | | 8·0049 (0·0914) | | | | | |  |
| Overall | | | 142 (40·9%) | | 162 (46·7%) | | 43 (12·4%) | 79 (20·9%) | 245 (64·8%) | | 54 (14·3%) | | 61 (19·5%) | | 216 (69·0%) | | 36 (11·5%) | | 53·474***^c^ (6·782e-11) |
| **Note.** ^a^The Chi-Squared have been computed within School SEC and without NA values for Adolescent SES. ^b^The Chi-Squared have been computed within Adolescent SES. ^c^Aggregated Chi-squared statistics between School SEC and PA Level. *p-value<0·05; **p-value<0·01; ***p-value<0·001. | | | | | | | | | | | | | | | | | | | |

| **Supplementary Table 7. PA Level by School SEC and Adolescent SES** | | | | | | | |  |
| --- | --- | --- | --- | --- | --- | --- | --- | --- |
| Variables | | School SEC | | | | | | Chi-squared (p-value)^b^ |
|  |  | Low | | Medium | | High | |  |
|  |  | PA Level | | PA Level | | PA Level | |  |
|  |  | Low | Medium\|High | Low | Medium\|High | Low | Medium\|High |  |
| ADOLESCENT- SES | LOW | 92 (43·0%) | 122 (57·0%) | 28 (25·9%) | 80 (74·1%) | 8 (27·6%) | 21 (72·4%) | 10·098** (0·006417) |
|  | MEDIUM | 25 (39·7%) | 38 (60·3%) | 25 (21·2%) | 93 (78·8%) | 22 (22·9%) | 74 (77·1%) | 8·027* (0·01807) |
|  | HIGH | 17 (34·0%) | 33 (66·0%) | 21 (15·3%) | 116 (84·7%) | 31 (17·1%) | 150 (82·9%) | 8·9985* (0·01112) |
|  | NA | 8 (40·0%) | 12 (60·0%) | 5 (33·3%) | 10 (66·7%) | 0 (0·0%) | 7 (100·0%) | 3·9438 (0·1392) |
| Chi-squared (p-value)^a^ | | 1·4087 (0·4944) | | 4·248 (0·1196) | | 2·4925 (0·2876) | |  |
| Overall | | 142 (40·9%) | 205 (59·1%) | 79 (20·9%) | 299 (79·1%) | 61 (19·5%) | 252 (80·5%) | 50·011***^c^ (1·381e-11) |
| **Note.** ^a^The Chi-Squared have been computed within school SEC and without NA values for adolescent SES. ^b^The Chi-Squared have been computed within Adolescent SES. ^c^Aggregated Chi-squared statistics between school SEC and Lunch Type. *p-value<0·05; **p-value<0·01; ***p-value<0·001. Medium\|High: Medium or High. | | | | | | | | |

| **Supplementary Table 8 Results from the full model without and with interaction in Medium\|High PA Level** | | | |
| --- | --- | --- | --- |
| Variables |  | Full Model without Interaction | Full Model with interaction |
| Age |  | 1·03 [0·91;1·17] | 1·03 [0·91;1·17] |
| Sex |  |  |  |
|  | Girls (ref.) |  |  |
|  | Boys | 1·98*** [1·47;2·67] | 1·98*** [1·47;2·68] |
| Adolescent SES | |  |  |
|  | Low (ref.) |  |  |
|  | Medium | 1·14 [0·78;1·68] | 1·09 [0·61;1·98] |
|  | High | 1·79** [1·20;2·68] | 1·46 [0·76;2·88] |
| School SEC | |  |  |
|  | Low (ref.) |  |  |
|  | Medium | 2·93*** [1·93;4·5] | 2·64*** [1·52;4·69] |
|  | High | 2·63*** [1·61;4·32] | 2·32 [0·96;6·10] |
| School Lunch | |  |  |
|  | Outside school (ref.) |  |  |
|  | At school | 0·77 [0·53;1·12] | 0·77 [0·53;1·12] |
| Household Composition | |  |  |
|  | Live with both parents (ref.) |  |  |
|  | Do not live with both parents | 0·69* [0·51;0·93] | 0·69* [0·51;0·94] |
|  | Number of Siblings (M, SD) | 1·05 [0·95;1·17] | 1·05 [0·95;1·16] |
| Adolescent SES x School SEC | |  |  |
|  | Medium Adolescent SES x Medium School SEC |  | 1·13 [0·48;2·67] |
|  | High Adolescent SES x Medium School SEC |  | 1·40 [0·55;3·51] |
|  | Medium Adolescent SES x High School SEC |  | 1·14 [0·36;3·44] |
|  | High Adolescent SES x High School SEC |  | 1·37 [0·43;4·16] |
| AIC |  | 1097·7 | 1105·1 |
| **Note.** Ref. : Reference category; *p-value<0·05; **p-value<0·01; ***p-value<0·001. AIC: Aikake Information Criterion. | | | |

| **Supplementary Table 9.** PA Level Odds-Ratio Contrast Analysis of School-SEC and Adolescent SES | | |
| --- | --- | --- |
| Within School | Adolescent-SES Contrasts |  |
| Low-SEC Schools |  | Estimate Differences |
|  | Low-Medium | 0·92 [0·36;2·32] |
|  | Medium-High | 0·75 [0·21;2·60] |
|  | Low-High | 0·68 [0·24;1·95] |
| Medium-SEC Schools |  |  |
|  | Low-Medium | 0·81 [0·30;2·21] |
|  | Medium-High | 0·60 [0·21;1·70] |
|  | Low-High | 0·49 [0·17;1·37] |
| High-SEC Schools |  |  |
|  | Low-Medium | 0·81 [0·18;3·67] |
|  | Medium-High | 0·62 [0·23;1·66] |
|  | Low-High | 0·50 [0·12;2·12] |
| Within SES | School-SEC Contrasts |  |
| Low-SES Adolescents |  | Estimate Differences |
|  | Low-Medium | 0·38* [0·16;0·92] |
|  | Medium-High | 1·14 [0·25;5·15] |
|  | Low-High | 0·43 [0·10;1·84] |
| Medium-SES Adolescents |  |  |
|  | Low-Medium | 0·34 [0·11;1·05] |
|  | Medium-High | 1·13 [0·39;3·28] |
|  | Low-High | 0·38 [0·11;1·26] |
| High-SES Adolescents |  |  |
|  | Low-Medium | 0·27* [0·08;0·95] |
|  | Medium-High | 1·16 [0·43;3·10] |
|  | Low-High | 0·31 [0·09;1·06] |
| **Note.** *p-value<0·05. | | |

| **Supplementary Table 10.** All significant crossed interaction effects | |
| --- | --- |
| School SEC, Adolescents SES | Odds-Ratio |
| LOW,LOW - HIGH,MEDIUM | 6·04** [1·77;28·62] |
| LOW,LOW – MEDIUM,HIGH | 2·51* [1·02;6·33] |
| MEDIUM,LOW - HIGH,MEDIUM | 6·71** [2·02;30·89] |
| MEDIUM,LOW - HIGH,HIGH | 2·34* [1·02;5·45] |
| LOW,MEDIUM - MEDIUM,HIGH | 4·46* [1·50;12·51] |
| LOW,MEDIUM - HIGH,HIGH | 3·75* [1·39;10·04] |
| HIGH,MEDIUM - LOW,HIGH | 0·07*** [0·01;0·31] |
| **Note.** *p-value<0·05; **p-value<0·01; ***p-value<0·001. | |

| **Supplementary Table 11.** Results from the univariable, full model without and with interaction to estimate BMI Z-Scores | | | | |
| --- | --- | --- | --- | --- |
| Variables |  | Univariable Analysis | Full Model without Interaction | Full Model with interaction |
| Age | | -0·08*** [-0·12;-0·03] | -0·12*** [-0·17;-0·07] | -0·12 [-0·17;-0·06] |
| Sex | |  |  |  |
|  | Girls (ref.) |  |  |  |
|  | Boys | -0·02 [-0·15;0·10] | 0·03 [-0·09;0·16] | 0·03 [-0·09;0·16] |
| Adolescent SES | |  |  |  |
|  | Low (ref.) |  |  |  |
|  | Medium | -0·28*** [-0·44;-0·12] | -0·16 [-0·33;0·00] | -0·05 [-0·32;0·23] |
|  | High | -0·35*** [-0·50;-0·21] | -0·18* [-0·34;-0·01] | -0·01 [-0·31;0·29] |
| School SEC | |  |  |  |
|  | Low (ref.) |  |  |  |
|  | Medium | -0·15* [-0·29;0·00] | -0·29** [-0·46;-0·11] | -0·17 [-0·41;0·07] |
|  | High | -0·47*** [-0·62;-0·32] | -0·50*** [-0·70;-0·29] | -0·35 [-0·74;0·04] |
| School Lunch | |  |  |  |
|  | Outside school (ref.) |  |  |  |
|  | At school | -0·16* [-0·28;-0·03] | 0·07 [-0·08;0·22] | 0·08 [-0·07;0·23] |
| Physical Activity | |  |  |  |
|  | Low (ref.) |  |  |  |
|  | Medium | -0·04 [-0·18;0·10] | 0·11 [-0·03;0·26] | 0·12 [-0·03;0·26] |
|  | High | -0·15 [-0·35;0·06] | -0·04 [-0·25;0·17] | -0·05 [-0·26;0·17] |
| Household Composition | |  |  |  |
|  | Live with both parents (ref.) |  |  |  |
|  | Do not live with both parents | 0·12 [0·00;0·25] | 0·08 [-0·05;0·21] | 0·08 [-0·05;0·21] |
|  | Number of Siblings (M, SD) | 0·07*** [0·03;0·11] | 0·03 [-0·01;0·08] | 0·04 [-0·01;0·08] |
| Adolescent SES x School SEC | |  |  |  |
|  | Medium Adolescent SES x Medium School SEC |  |  | -0·15 [-0·52;0·23] |
|  | High Adolescent SES x Medium School SEC |  |  | -0·33 [-0·71;0·06] |
|  | Medium Adolescent SES x High School SEC |  |  | -0·32 [-0·80;0·17] |
|  | High Adolescent SES x High School SEC |  |  | -0·23 [-0·72;0·25] |
| AIC | |  | 2771·22 | 2774·09 |
| Adj-R² | |  | 0·06 | 0·06 |
| **Note.** Ref. : Reference category; *p-value<0·05; **p-value<0·01; ***p-value<0·001. AIC: Aikaike Information Criterion. Adj-R²: Adjusted R-squared. | | | | |
