# Supplementary material for: Overweight and obesity by school socioeconomic composition and adolescent socioeconomic status: a school-based study
Source: BMC Public Health. 2021 Oct 11;21:1837. doi: 10.1186/s12889-021-11752-2 (PMC8507235; doi:10.1186/s12889-021-11752-2)
Supplement: Supplementary file 2 — Additional file 2 Questionnaire. Adolescent students' physical and sport activity. [file 12889_2021_11752_MOESM2_ESM.pdf]

Questionnaire

Adolescent students' physical and sport activity

The goal of this survey is to inform us about your **physical and sports activities**. In answering this survey **spontaneously** and **sincerely**, you will bring precious informations to researchers at the Faculty of Sports Sciences, Marseille. Your answer will be **anonymous** and used only for **scientific purposes**. No commercial use is warranted.

We thank you for your participation.

1. Age ☐ **2.** Height ☐ **3.** Weight ☐ **4.** Number of Siblings ☐

5. Sex Female ☐ Male ☐ **6.** School Board Full board ☐ ½ board ☐ No board ☐

7. You live with your parents ☐ your father ☐ your mother ☐ alternating ☐ other(s) ☐

8. Father's and Mother's Occupation (*tick the correct answer(s)*)

	Mother	Father
Farmer		
Merchant, artisan, company director		
Intellectual occupation (engineer, doctor/physician, etc.)		
Intermediate occupation (teacher, nurse, technician, etc.)		
Salaried employee		
Manual worker		
Retired		
Unemployed		
At home		
Other (precise) …………………………………………………………		

7. In a typical week, on how many days do you bicycle for at least 10 minutes continuously to get to and from places?

☐

8. How much time do you spend bicycling for travel on a typical day? ☐ Hours ☐ Minutes

9. Apart from physical education classes at school, do you do sports? *(If not, the questionnaire ends there)*

Yes ☐ No ☐

7. In a typical week, on how many days do you do sports ? ☐

8. In a typical week, how much time do you spend doing sports ? ☐ Hours ☐ Minutes

9. What sport do you do the most (main sport) ? *(Only one answer)* ☐

10. You play your main sport… (*multiple answers possible)* ☐ club ☐ outside-club

11. How long have you been playing your main sport ? ☐ year(s) and ☐ month(s)

12. What is your level of competition in your main sport ?

I do not play in competition	
Local/community level	
Regional/state level	
National level	
International level	
Official high-level athletes recognized by the Ministry of Sports	

13. In a typical week, on how many days do you do your main sport ? ☐

14. In a typical week, how much time do you spend doing your main sport ? ☐ Hours ☐ Minutes
